# Primary breast cancer biomarkers based on glycosylation and extracellular vesicles detected from human serum

**DOI:** 10.1002/cnr2.1540

**Published:** 2021-08-22

**Authors:** Joonas Terävä, Alejandra Verhassel, Orsola Botti, Md. Khirul Islam, Janne Leivo, Saara Wittfooth, Pirkko Härkönen, Kim Pettersson, Kamlesh Gidwani

**Affiliations:** ^1^ Department of Biochemistry University of Turku Turku Finland; ^2^ Institute of Biomedicine and FICAN West Cancer Research Laboratory University of Turku Turku Finland

**Keywords:** breast neoplasms, glycosylation, in vitro diagnostics, mucins, tetraspannin‐30

## Abstract

**Background:**

Breast cancer is a very common cancer that can be severe if not discovered early. The current tools to detect breast cancer need improvement. Cancer has a universal tendency to affect glycosylation. The glycosylation of circulating extracellular vesicle‐associated glycoproteins, and mucins may offer targets for detection methods and have been only explored in a limited capacity.

**Aim:**

Our aim was to develop an approach to detect the aberrant glycosylation of mucins and extracellular vesicle‐associated glycoproteins from human sera using fluorescent nanoparticles, and preliminarily evaluate this approach for the differential diagnosis of breast cancer.

**Methods and results:**

The assay involved immobilizing glycosylated antigens using monoclonal antibodies and then probing their glycosylation by using lectins and glycan‐specific antibodies coated on Eu^+3^‐doped nanoparticles. Detection of mucin 1 and mucin 16 glycosylation with wheat germ agglutinin, and detection of the extracellular vesicle‐associated CD63 were found to have better diagnostic ability for localized breast cancer than the conventional assays for mucin 1 and mucin 16 based tumor markers when the receiver operating characteristics were compared.

**Conclusions:**

These results indicate that successful differential diagnosis of primary breast cancer may be aided by detecting cancer‐associated glycosylation of mucin 1 and mucin 16, and total concentration of CD63, in human serum.

**Abbreviations**
AAL: *Aleuria aurantia* lectin
AUC: area under curve
BrCa: breast cancer
CA: cancer antigen
CD63: tetraspannin‐30
CEA: carcinoembryonic antigen
Con‐A: Concanavalin A
DC‐sign: Dendritic Cell‐Specific Intercellular adhesion molecule‐3‐grabbing non‐integrin
EDC: N‐(3‐dimethylaminopropyl)‐N′‐ethylcarbodiimide
ER−: estrogen receptor negative
Eu + 3‐NP: Eu + 3‐doped nanoparticles
EV: extracellular vesicle
FBS: fetal bovine serum
GBP: glycan‐binding proteins
GalNAc: N‐acetylgalactosamine
GlcNAc: N‐acetylglucosamine
HER2−: human epidermal growth factor receptor 2 negative
IA: immunoassay
LacNAc: N‐acetyllactosamine
LTL: *Lotus tetragonolobus* lectin
MAA: *Maackia amurensis* agglutinin
MES: 2‐(N‐morpholino)ethanesulfonic acid
MGL: macrophage galactose binding lectin
MUC1: mucin 1
MUC16: mucin 16
PR−: progesterone receptor negative
ROC: Receiver Operating Characteristics
S/B: signal‐to‐background ratio
SA: streptavidin
SBA: soybean agglutinin
sulfo‐NHS: N‐hydroxysulfosuccinimide
UEA: *Ulex Europaeus* agglutinin
WGA: wheat germ agglutinin

## 1 INTRODUCTION

Breast cancer (BrCa) is the second most diagnosed cancer and the leading cause of cancer death in women, with an estimated number of 627 000 cancer deaths in 2018. The incidence of BrCa is increased in countries with high human development index but even in other countries, the incidence rates are on the rise. The underlying cause for the differences in incidence rates correlating with human development index, is most likely due to many social and economic factors.^1^ Small primary tumors are treatable with surgery and have a high relative 5 year survival rate; even 100% for ≤1 cm tumors.^2^ That is why early detection is important and screening for BrCa can reduce mortality. Detecting cancer early can also reduce costs in healthcare as early stage cancer does not require continued expensive treatment. Mammography‐based screening is the main modality of screening used for BrCa, but it has its limitations. It has been reported that overdiagnosis and consequent overtreatment, false‐positive biopsies and radiation‐induced BrCa are the main harms of mammography‐based screening.^3^ Dense breast tissue also decreases the sensitivity and accuracy of mammographic screening.^4^ The breasts of premenopausal women are generally denser than those of older women.^5^

On average, women from industrialized countries experience menopause at between 50 and 52 years of age, with differences related to ethnicity, demographics, and lifestyle.^6^ About 31% of women diagnosed with BrCa are under the age of 50.^1^ Although BrCa is generally more uncommon in premenopausal women, the tumor size is on average larger and the tumor stage is more advanced than tumors diagnosed in postmenopausal women, making the prognosis generally worse and the survival rate lower.^7^ This could also to some extent be due to the fact that it is commonly recommended for women to start undergoing mammographic screenings after the age of 45.^8^ Estrogen and progesterone receptor negative (ER^−^, PR^−^) and human epidermal growth factor receptor 2 negative (HER2^−^), that is, triple negative BrCa has higher incidence in premenopausal women compared to older women.^9^ Premenopausal BrCa is suggested to have distinct biological characteristics that differentiate it well from postmenopausal BrCa and contribute to its poor prognosis.^10^ These differences highlight the importance of using a cohort of BrCa samples from the same age group (premenopausal/postmenopausal) in research for early diagnostics and new screening methods.

Currently, no serological biomarkers are used for BrCa diagnosis or screening due to the lack of sensitivity and specificity. The only FDA‐approved serum markers for BrCa are based on mucin 1 (MUC1) epitopes (CA15‐3, CA27.29) and are used for monitoring the disease.^11^ However, other glycoprotein markers, such as cancer antigen 125 (CA125/mucin 16), cancer antigen 19–9 (CA19‐9), and carcinoembryonic antigen (CEA), may also be elevated in some BrCa patients.^12^ Presently, serological biomarkers have very limited utility for aiding clinical decisions concerning BrCa diagnosis or treatment.

Protein glycosylation is a co‐ and post‐translational process that is readily influenced by the conditions of the cell. It has been extensively reported that glycosylation is affected by malignant transformation, and the microevolutionary processes of the cancer microenvironment guide the resulting glycovariant proteins toward a malignant phenotype. These glycovariants may contribute to all hallmarks of cancer and be found in bodily fluids, such as blood and urine.^13^ These kinds of molecules may offer sensitive and minimally invasive ways to help diagnose early stage cancers if suitable methodology for detecting them is introduced. Cancer‐associated glycoproteins may also be attached to the surfaces of extracellular vesicles (EVs), such as exosomes.^14^ Especially tetraspanin‐30 (CD63), and its aberrant glycosylation, has been associated with BrCa cell malignancy.^15^ The CD63 glycoprotein is abundantly expressed on different subtypes of EVs and can be detected directly from human blood.^16^

Currently, methods for early detection of BrCa are lacking, but there are several leads for new tumor markers, including CA125, CA15‐3, and CD63 glycovariants. We used a cost‐effective and high sensitivity immunoassay‐type approach to screen for glycovariants of these glycoproteins. We found that glycovariants of CA15‐3 defined by wheat germ agglutinin (WGA), anti‐T antibody, and anti‐Tn antibody, glycovariants of CD63 defined by *Ulex Europaeus* agglutinin (UEA), and glycovariants of CA125 defined by WGA and anti‐T antibody were elevated in BrCa cell lines compared to a non‐malignant breast cell line. Assays measuring the glycovariant markers and the total CD63 in serum performed significantly better in differentiating BrCa patients from healthy individuals than the conventional CA125 and CA15‐3 assays. The cancerous samples were mostly early stage BrCa samples but no histopathological analysis were performed on them so connecting breast cancer subtypes to certain glycovariants was not possible. The results imply that there is room for improvement upon the conventional tumor markers for differential diagnosis, although the results should be verified on a larger cohort.

## 2 MATERIALS AND METHODS

### 2.1 Clinical samples

Serum samples (*N* = 18) from healthy female volunteers were purchased from TUAS Lab (Turku, Finland) and serum samples (*N* = 16) from premenopausal BrCa patients were purchased from Discovery Life Sciences, Inc. (Huntsville, Alabama). The age of healthy volunteers ranged from 31 to 49 years, and the age of the cancer patients from 33 to 50 years. The median of ages was 38.5 and 42 years for the controls and cases, respectively. All the cancer samples had been taken before the start of cancer treatment, all were from Caucasian females, and none of them had a history of benign disease. Two of the samples were from stage I, seven from stage II, five from stage III, and two from stage IV BrCa patients.

### 2.2 Materials, reagents, and equipment

The 96‐well microtiter plates coated with streptavidin (SA plates, product #: 41‐07TY), assay wash buffer (product #: 42‐01TY), and RED assay buffer (product #: 42‐02TY) were purchased from Kaivogen Oy (Turku, Finland). From Fujirebio Diagnostics, we acquired the anti‐CA125 antibody Ov197 and anti‐CA15‐3 antibody Ma552 as well as the conventional assay kits for CA125 and CA15‐3. The anti‐CD63 antibody was purchased from R&D Systems (Minneapolis, Minnesota). The plate washer (Delfia PlateWash 1296–026), plate shaker (Delfia PlateWash 1296‐026), and VictorX4™ fluorimeter were from Wallac Oy (Turku, Finland). The reagents for cell line cultures were Gibco brand of Thermo Fisher Scientific (Waltham, Massachusetts) except for glutamine, which was Ultraglutamine from Lonza (Basel, Switzerland), and phosphate buffered saline which was from GE Healthcare (Chicago, Illinois).

### 2.3 Cell cultures

The human BrCa cell lines SKBR3 (RRID:CVCL_0033) and T47D (RRID:CVCL_0553) were cultured in Dulbecco's Modified Eagle Medium (DMEM), supplemented with 10% inactivated fetal bovine serum (FBS), 1% glutamine and 1% penicillin–streptomycin. The human BrCa cell line MCF7 (RRID:CVCL_0031) was cultured in DMEM, supplemented with 10% inactivated FBS, 1% glutamine, 1% penicillin–streptomycin and 10 μg∙ml^−1^ insulin (insulin, human recombinant, zinc solution). The immortalized human breast epithelial cell line MCF10A (RRID:CVCL_0598)) was cultured in advanced DMEM /F12 medium, supplemented with 20% inactivated FBS, 1% glutamine, 1% penicillin–streptomycin and 10 μg/mL insulin (human recombinant, zinc solution). The cells were cultured at 37°C under 5% CO_2_. When the cells reached the confluence of approx. 60%, the medium was collected and centrifuged for 3 min at 161*g*. The supernatant, that is, spent medium, was collected and stored at −80°C. The spent medium was then concentrated 4–6 times with Vivaspin Turbo 15 filter (Sartorius Stedim Lab Ltd, Stonehouse, UK) and stored at −20°C. The identity of all cell lines were verified using short tandem repeat (STR) analysis by IdentiCell service (Aarhus, Denmark) and the cells have been verified to be free of mycoplasma.

### 2.4 Glycan‐binding reporter molecules

The molecules used for coating the Fluoro‐Max™ carboxylate‐modified Eu^+3^‐doped nanoparticles (Eu^+3^‐NPs) are presented in Table 1. The coating and usage of Eu^+3^‐NPs as bioconjugated reporter molecules has been described previously.^17^ The conjugation of the GBPs was performed using N‐(3‐dimethylaminopropyl)‐N′‐ethylcarbodiimide(EDC; Merck, Darmstadt, Germany)‐induced crosslinking, stabilized with N‐hydroxysulfosuccinimide (sulfo‐NHS; Sigma‐Aldrich, St. Louis, Missouri). The procedure for conjugating the molecules has been described previously^18^ except for *Aleuria aurantia* lectin (AAL), Dendritic Cell‐Specific Intercellular adhesion molecule‐3‐grabbing non‐integrin (DC‐sign), *Lotus tetragonolobus* lectin (LTL), *Maackia amurensis* agglutinin (MAA), soybean agglutinin (SBA), and UEA. These lectins were coated on particles activated with a 100 times molar excess of EDC and sulfo‐NHS compared to the carboxyl groups on the particles. The particles were activated by incubating with the excess of EDC and sulfo‐NHS in 50 mM 2‐(N‐morpholino)ethanesulfonic acid (MES) buffer (pH 6.1) in Thermomixer® (Eppendorf AG, Hamburg, Germany) for 15 min (700 rpm, 37 °C). The Eu^+3^‐NPs were resuspended like in the original protocol in under 20 min into the buffer each lectin came supplied in, or was recommended to be suspended in, and 50–100 μg of each lectin, and NaCl to a final concentration of 100 mM, was added. The reaction was then incubated overnight and continued as the original protocol continues after the recognition molecule conjugation.

**TABLE 1:** Lectins and antibodies used

Abbreviation	Full name	Major carbohydrate binding specificity	Vendor
AAL	Aleuria aurantia lectin	α1‐6 linked fucose	Vectorlabs^a^
BPL	*Bauhinia purpurea* lectin	galactosyl (β‐1,3)GalNAc structures	Vectorlabs^a^
Con‐A	Concanavalin A	α‐d‐mannosyl and α‐d‐glucosyl residues branched α‐mannosidic structures	Vectorlabs^a^
DBA	*Dolichos biflorus* agglutinin	α‐linked GalNAc	Vectorlabs^a^
DC‐sign	Dendritic Cell‐Specific Intercellular adhesion molecule‐3‐Grabbing Non‐integrin	Nonsialylated Lewis antigens and high mannose‐type structures	R&D Systems
Dectin 1	Dendritic cell‐associated C‐type lectin‐1	β‐1,3‐linked and β‐1,6‐linked glucans	R&D Systems^b^
Dectin 2	Dendritic cell‐associated C‐type lectin‐2	α‐mannose residues	R&D Systems^b^
DSL	*Datura Stramonium* Lectin	(β‐1,4) linked GlcNAc oligomers	Vectorlabs^a^
E‐sel	E‐selectin	Sialylated Lewis^x^	R&D Systems^b^
FCN‐1	Ficolin‐1	sialic acid and acetylated moieties	R&D Systems^b^
FCN‐2	Ficolin‐2	multiple, including N‐glycans, β‐1,3‐glucans and neauramidase	R&D Systems^b^
FCN‐3	Ficolin‐3	acetylated polysaccharides, D‐fucose and galactose	R&D Systems^b^
Gal‐3	Galectin‐3	terminal or α2–3‐sialylated LacNAc, GalNAc, galactose	R&D Systems^b^
Gal‐4	Galectin‐4	SO3‐3galactoseβ1‐3GalNAc pyranoside	R&D Systems^b^
GSL‐1b	Griffonia Simplicifolia Lectin I	α‐galactose residues	Vectorlabs^a^
GSL‐2	Griffonia Simplicifolia Lectin II	α‐ or β‐linked GlcNAc residues on the nonreducing terminal of oligosaccharides	Vectorlabs^a^
HPA	*Helix pomatia* agglutinin	GalNAc (Tn antigen)	Vectorlabs^a^
Jacalin	Artocarpus integrifolia (Jackfruit) lectin	O‐glycosidically linked oligosaccharides (galactosyl (β‐1,3) GalNAc or T antigen)	Vectorlabs^a^
LCA	*Lens culinaris* agglutinin	α‐linked Fucose‐N‐acetylchitobiose	Vectorlabs^a^
LEL	*Lycopersicon esculentum* lectin	[GlcNAc]1–3, GlcNAc	Vectorlabs^a^
LTA	*Lotus tetragonolobus* agglutinin	α‐linked L‐fucose containing oligosaccharides	Vectorlabs^a^
MAA	*Maackia amurensis* lectin	N‐linked glycans containing the trisaccharide Sia‐3galactoseβ1‐4GlcNAc	Vectorlabs^a^
MGL	Macrophage galactose‐type lectin	Terminal α‐or β‐linked GalNAc	R&D Systems^b^
MSLN	Mesothelin	N‐glycans on CA125	R&D Systems^b^
NPA	Narcissus pseudonarcissusagglutinin	α‐mannose residues	Vectorlabs^a^
PHA‐E	*Phaseolus vulgaris* agglutinin‐erythroagglutinin	Bisecting GlcNAc	Vectorlabs^a^
PSA	*Pisum sativum* agglutinin	α‐Mannose	Vectorlabs^a^
PTA	*Psophocarpus tetragonolobus* agglutinin	GalNAc	EY Labs^e^
RCA	*Ricinus communis* agglutinin	Galactoseβ1‐4GlcNAc	Vectorlabs^a^
RPL Fuc‐1	Recombinant prokaryotic lectin Fuc‐1	α‐linked Fucose	Glycoselect^d^
RPL Man‐1	Recombinant prokaryotic lectin Man‐1	mannose	Glycoselect^d^
RPL Man‐2	Recombinant prokaryotic lectin Man‐2	Terminal α‐mannose	Glycoselect^d^
SBA	Soybean agglutinin	Terminal α‐or β linked GalNAc	Vectorlabs^a^
Siglec‐15	Sialic acid‐binding Ig‐like lectin 15	sialic acid‐containing structures	R&D Systems^b^
Siglec‐2	Sialic acid‐binding Ig‐like lectin 2	sialic acid‐containing structures	R&D Systems^b^
Siglec‐3	Sialic acid‐binding Ig‐like lectin 3	sialic acid‐containing structures	R&D Systems^b^
SNA	*Sambucus nigra* agglutinin	Sialic acid α2‐6galactose	Vectorlabs^a^
SSA	*Salvia sclarea* agglutinin	Tn antigen	EY Labs^e^
SSL‐1	Staphylococcal superantigen‐like 1	Matrix metalloproteinases	University of Turku
SSL‐10	Staphylococcal superantigen‐like 10	Immunoglobulin G	University of Turku
SSL‐5	Staphylococcal superantigen‐like 5	sialic acid	University of Turku
STL	*Solanum tuberosum* lectin	oligosaccharides containing GlcNAc and N‐acetylmuramic acid	Vectorlabs^a^
T mAb	Anti‐T antigen monoclonal antibody	Galactoseβ3GalNAcα1‐O‐Ser/Thr	SBH Sciences^c^
TJA II	Trichosanthes japonica agglutinin II	Fucoseα1‐2galactose and β‐GalNAc	Vectorlabs^a^
Tn mAb	Anti‐Tn antigen monoclonal antibody	GalNAcα1‐O‐Ser/Thr	SBH Sciences^c^
UEA	*Ulex europaeus* agglutinin	Fucoseα1‐2galactose	Vectorlabs^a^
WFL	*Wisteria floribunda* lectin	GalNAc	Vectorlabs^a^
WGA	Wheat germ agglutinin	Terminal GlcNAc or chitobiose	Vectorlabs^a^
VVL	*Vicia villosa* lectin	Terminal α‐or β‐linked GalNAc (Tn antigen)	Vectorlabs^a^

Abbreviations: GalNAc, N‐acetylgalactosamine; GlcNAc, N‐acetylglucosamine; LacNAc, N‐acetyllactosamine.

^a^ Burlingame, California.

^b^ Minneapolis, Minnesota.

^c^ Natick, Massachusetts.

^d^ Dublin, Ireland.

^e^ San Mateo, California.

### 2.5 Screening for glycovariant biomarkers

All incubations were performed at room temperature and all washes were done using Kaivogen wash buffer diluted to 1× concentration. All assays were done in triplicates. The antibodies against CA125, CA15‐3, and CD63 were biotinylated with 40 times molar excess of biotin isothiocyanate (University of Turku, Turku, Finland) in 50 mM sodium carbonate buffer (pH 9.8) by incubating for 4 h covered from light. The excess unreacted biotin isothiocyanate was removed with NAP‐5 and NAP‐10 gel filtration columns (GE Healthcare), twice. The biotinylated antibodies were stored in TSA‐BSA (Tris–HCl, pH 7.75; 150 mM NaCl; 0.5 g/L NaN_3_; 1 g/L BSA) at +4°C. The SA plates were washed once and 100 ng of biotinylated anti‐CA125, and anti‐CD63 antibodies and 60 ng of biotinylated anti‐CA15‐3 were added in 30 μL volume into separate wells and incubated in RED assay buffer for 45 min. The wells were washed twice and 22.5 μL of RED assay buffer was added, followed by the addition 7.5 μL DMEM or concentrated spent cell culture medium from each of the cultured cell lines. The plates were incubated for 1 h in slow shaking and washed four times. Then, 10^7^ glycan‐binding reporter molecules were added per well in 30 μL of RED assay buffer supplemented with 6 mM CaCl_2_ and 6 mM MgCl_2_. With the anti‐CA15‐3 antibody, 49 different reporter molecules were used, and with the anti‐CA125 and anti‐CD63 antibody 27 different reporter molecules were used. After a 90‐min incubation in slow shaking, the wells were washed four times and the time‐resolved fluorescence of Eu was measured with VictorX4 fluorometer (*λ*_ex_ = 340 nm, *λ*_em_ = 615 nm).

### 2.6 Assays on human serum

The assays on human serum samples were performed essentially like in the screening for glycovariant tumor markers. The exceptions were that 80 ng of each of the biotinylated antibodies were used for the immobilization to the streptavidin plate, and 1.5 μL of serum was added into 28.5 μL of RED assay buffer for CA15‐3 and CA125 glycovariant assays and 6 μL of serum into 24 μL RED assay buffer was added for CD63 glycovariant assays. TSA‐BSA was used as a blank. The CD63 glycovariant assay was also performed using Eu^+3^‐NPs coated with anti‐CD63 antibody. The concentration of CA125 and CA15‐3 was also measured using Fujirebio Diagnostics enzyme immunoassay kits according to the manufacturer's instructions and using all optional steps.

### 2.7 Statistical analyses

The glycovariant tumor marker screening results were plotted using RStudio^19^ software with ggpubr^20^ and ggplot2^21^ R packages. The concentrations from serum sample measurements were calculated and the box plots were plotted using Origin 2016^22^ software. The statistical differences (*α* = 0.05) between measured human serum concentrations of controls and cases were evaluated using the Wilcoxon rank sum test in RStudio.^19^ Logistic regression probabilities for BrCa using the assay measurements as classifiers were calculated using RStudio.^19^ Based on the probabilities, Receiver Operating Characteristics (ROC) and the ROC area under curve (AUC) were calculated and plotted with the pROC package^23^ in RStudio^19^ and the AUCs were compared with the same package using the bootstrap method. All p‐values were corrected for multiple testing using the Benjamini‐Hochberg method^24^ in Rstudio.^19^

## 3 RESULTS

### 3.1 Glycovariant biomarker screening

**FIGURE 1:**
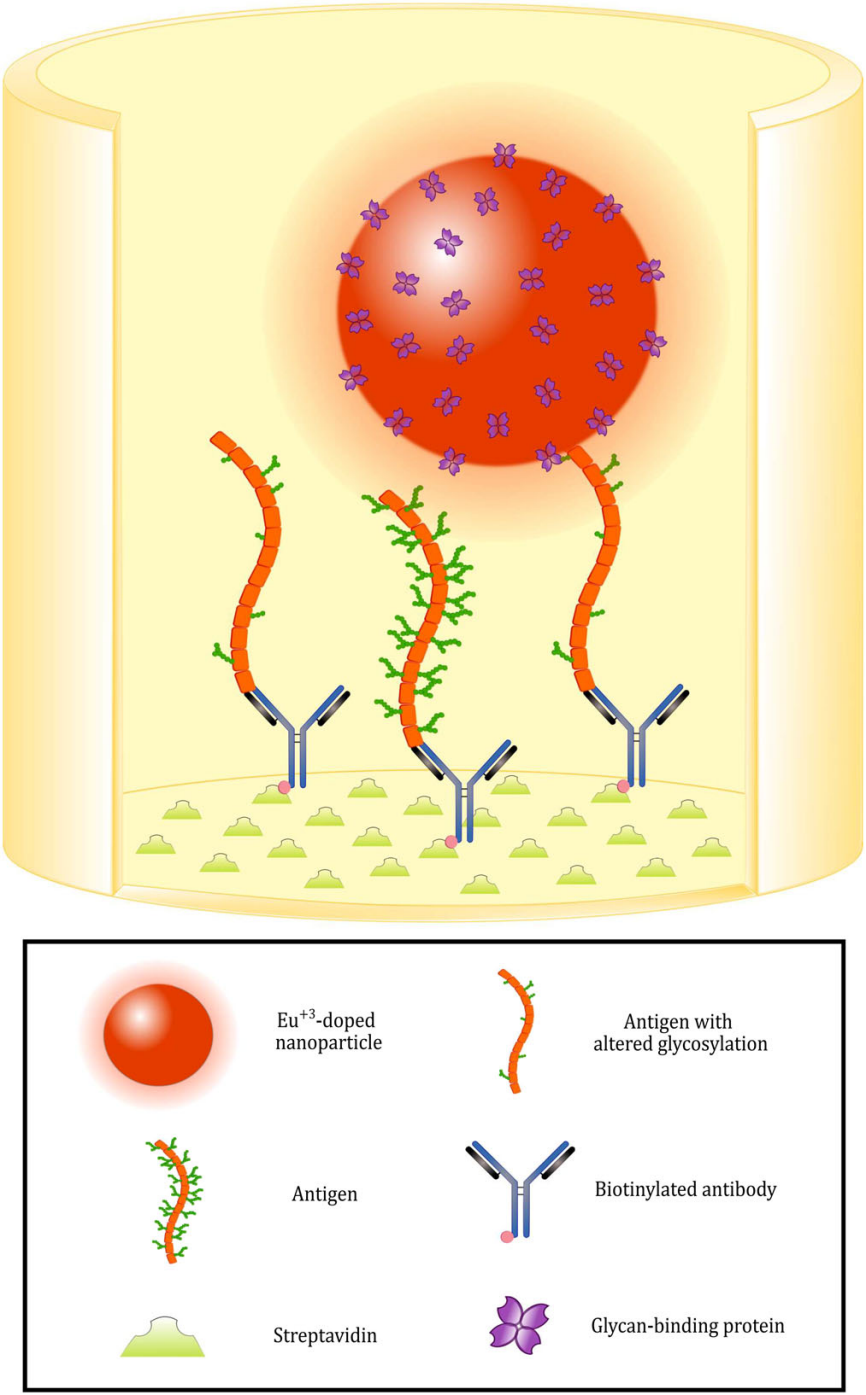
Schematic representation of the glycovariant assay principle. The biotinylated capture antibodies are immobilized on the surface of streptavidin‐coated yellow microtiter wells. The target antigen is then recognized by the capture antibody. In the final step, Eu^+3^‐doped nanoparticles coated with glycan‐binding proteins (lectins or antibodies) are added to the wells: the proteins coated on their surface recognize altered glycans on the cancer‐related target antigen. Between each step, the wells are washed to remove the unbound fraction of assay components. The measurement of time‐resolved fluorescence is performed on a spot on the bottom of each well

Antigens CA125, CA15‐3, and CD63 from three different BrCa cell lines, MCF7, SKBR3, and T47D, and the breast epithelial cell line MCF10A were immobilized from their concentrated spent media and their binding with glycan‐specific reporter molecules was determined using the assay depicted in Figure 1.

**FIGURE 2:**
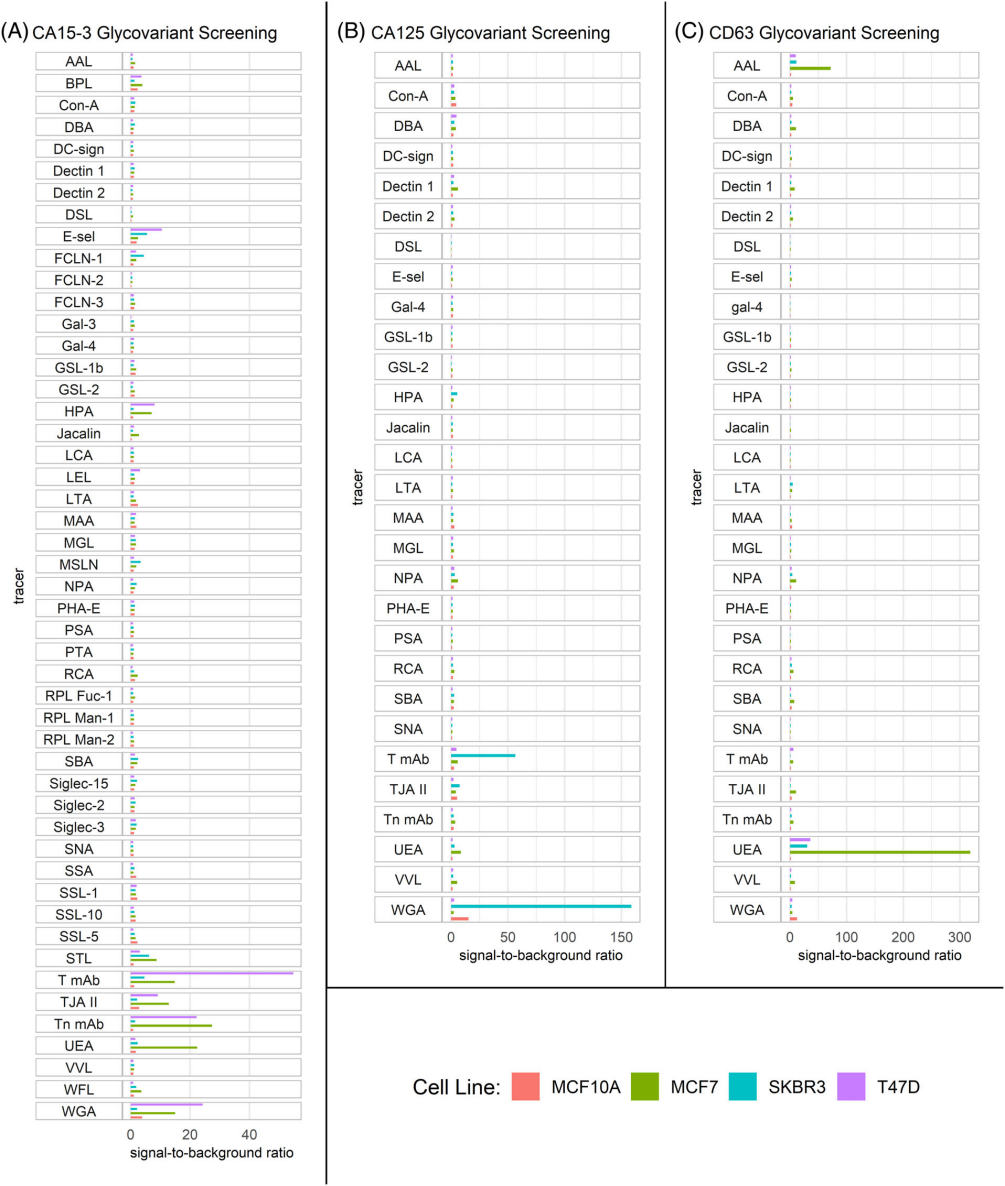
Glycovariant biomarker screening results. Signal‐to‐background ratios (S/B) from immobilizing antigens from breast cancer cell lines MCF7, SKBR3, and T47D, and breast epithelial cell line MCF10A and detecting with glycan‐specific fluorescent reporter molecules. (A) CA15‐3, (B) CA125, and (C) CD63 antigen glycovariant screening. Notice the different scale of x‐axes in (A), (B), and (C)

A signal‐to‐background ratio (S/B) of at least 15.0 was considered as an indication of significant binding of the glycan‐specific reporter molecule to the immobilized antigens. With immobilized CA15‐3, the binding of anti‐T antibody, anti‐Tn antibody, *N*‐acetylglucosamine (GlcNAc)‐binding wheat germ agglutinin (WGA), and fucose‐binding *Ulex europaeus* agglutinin (UEA) were elevated significantly. With CA125, significantly elevated binding was observed using WGA, and anti‐T antibody, while with immobilized CD63, especially UEA, and *Aleuria aurantia* lectin (AAL) bound effectively. The S/B from the antigens with each of the reporter molecules are presented in Figure 2.

Anti‐T antibody, anti‐Tn antibody, and WGA all bound to CA15‐3 from T47D with S/Bs of 54.8, 22.2, and 24.2, respectively. CA15‐3 from MCF7 was bound by anti‐Tn antibody, UEA, and WGA with S/Bs of 27.4, 22.4, and 15.0, respectively. Anti‐T antibody and WGA bound to CA125 from SKBR3 cell line with S/Bs of 56.6, and 158.6, respectively. WGA also bound to CA125 from MCF10A producing a S/B of 15.3. All the BrCa cell lines seemed to produce UEA‐reactive CD63 or CD63 complexes but MCF7 produced the highest S/B of 318.3, while T47D, and SKBR3 produced a S/B of 35.8 and 30.2, respectively. AAL, which is also a fucose‐binding lectin like UEA, bound with immobilized CD63 from MCF7 cell line with a S/B of 71.9.

### 3.2 Glycovariant assays on clinical samples

**FIGURE 3:**
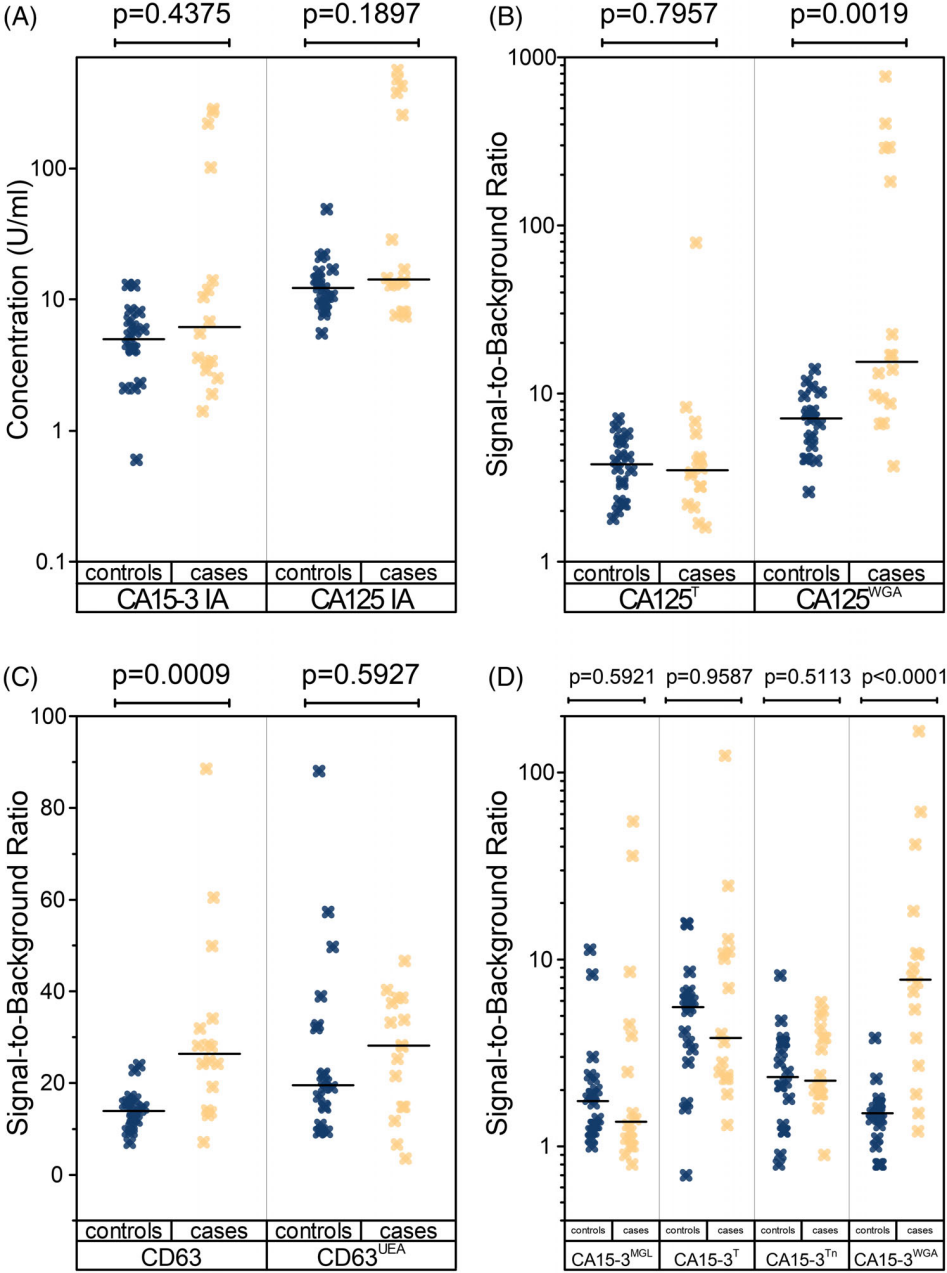
Conventional and glycovariant assay results from clinical samples. Box plots of the (A) established conventional CA125 (CA125 IA), and CA15‐3 (CA15‐3 IA) assays, (B) CA125 glycovariant assays, (C) CD63 (CD63 IA), and CD63^UEA^ assays, and (D) CA15‐3 glycovariant assays. The uncorrected *p*‐values on top of each plot are calculated with the Wilcoxon rank sum test. The significant p‐values (*α* = 0.05) remained significant after correction for multiple comparisons

The glycan‐binding proteins (GBPs) conjugated on the reporter molecules that displayed significant binding with an immobilized antigen were used to assay clinical samples. These assays were denominated as antigen^GBP^, for example, CA125^WGA^. In these cases, anti‐T antibody, and anti‐Tn antibody are abbreviated as T, and Tn, respectively. The conventional immunoassays for CA125 and CA15‐3 (CA125 IA, CA15‐3 IA) were also performed as well as an assay on immobilized CD63 using an anti‐CD63‐conjugated fluorescent nanoparticle (CD63 IA). The CD63^AAL^ assay was not performed because AAL binds fucose as does UEA and did not produce as high S/Bs. The glycovariant assay for CA15‐3 bound by macrophage galactose binding lectin (MGL) was also performed due to previous results from a metastatic BrCa patient cohort.^18^ The results from these assays is depicted in Figure 3 along with the p‐values from the Wilcoxon rank sum test. Significant differences (*α* = 0.05) between the case and control groups were observed for CD63 IA (*p* = .0009), CA125^WGA^ (*p* = .0019), and CA15‐3^WGA^ (*p* < .0001) assay measurements. After correction for multiple comparisons, the *p*‐values were .0046, .0063, and .0004 for CD63 IA, CA125^WGA^, and CA15‐3^WGA^, respectively.

**FIGURE 4:**
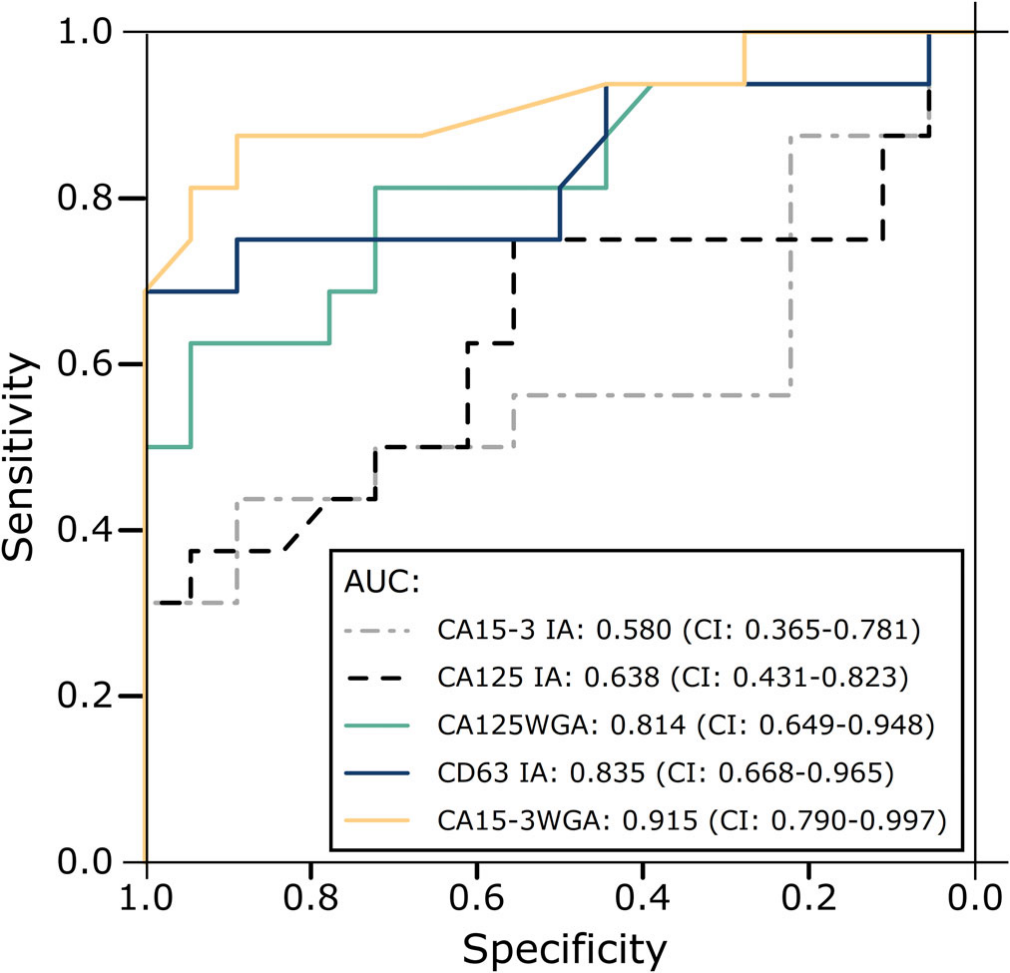
Receiver Operating Characteristics of the conventional, and glycovariant assays. The concentrations of the conventional biomarkers (CA125 IA and CA15‐3 IA), and the signal‐to‐background ratios of the experimental assays were used for classifying the healthy controls (*n* = 18) and breast cancer patients (*n* = 16). The 95% confidence interval (CI) is noted in parentheses after each area under the curve (AUC) value. The conventional CA125 and CA15‐3 assay (CA125 IA, CA15‐3 IA) are plotted in addition to the experimental wheat germ agglutinin‐reactive glycovariant assays for CA125 (CA125^WGA^) and CA15‐3 (CA15‐3WGA), and the experimental CD63 immunoassay (CD63 IA)

The assays which produced significant differences between the control and case groups, and the conventional assays, were analyzed for Receiver Operating Characteristics (ROC). The area under the ROC curve (AUC) was used to determine the overall clinical performance (Figure 4).

The AUCs of the experimental assays were compared to the AUCs of the conventional assays using the bootstrap method with 10 000 replicates. Significant differences (*α* = 0.05) were found when comparing CA125 IA with CA15‐3WGA (*p* = .0089), and CA15‐3 assay with CA125WGA (*p* = .0204) and CA15‐3WGA (*p* = .0004) assays. The comparisons remained significant after correction for multiple testing.

**FIGURE 5:**
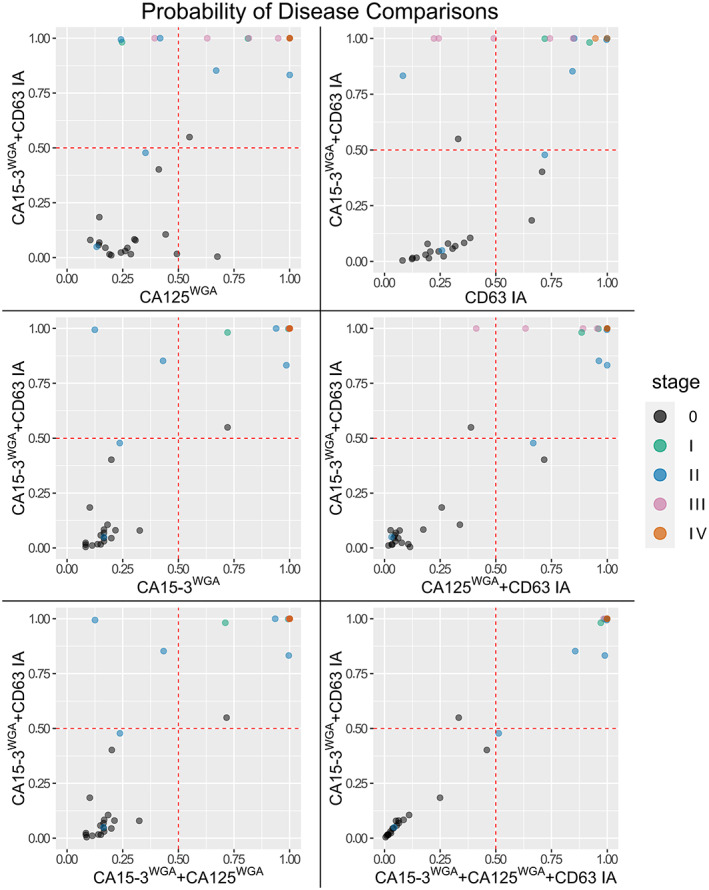
Comparisons of disease probability generated using logistic regression. The probabilities were generated using individual or multiple assays' results from the entire cohort as the classifying variables. The cut‐off of 0.5 is highlighted with dashed red line and the data points are colored according to the stage of the disease with stage 0 representing the healthy controls. The data points have transparency, which distorts the color of individual data points

Logistic regression models for the combination of the experimental assays were also generated and compared in Figure 5. The combination of CA15‐3WGA and CD63 IA yielded the best AUC of the two‐assay combinations with an AUC of 0.965 which is very close to the AUC produced by the combination of all experimental assays, which was 0.969.

## 4 DISCUSSION

BrCa is the first cause of cancer‐related deaths in women worldwide. Its incidence rates are constantly rising and its aggressiveness is greater in younger, premenopausal women. Diagnosing aggressive BrCa at an early stage would greatly benefit the individuals affected and lower the treatment costs. Screening methods, mainly mammography, have been employed to aid early detection but they come with their downsides such as overdiagnosis and overtreatment, and costs that may exceed the financial benefits achieved. Circulating biomarkers have great potential for early detection of cancer but that potential is yet to be realized. Circulating CA15‐3 levels are used for monitoring BrCa patients and CA125 concentrations have been previously found to be elevated in BrCa patients' blood. These proteins are known to be highly glycosylated and their glycosylation to be aberrant in BrCa.

EVs, such as exosomes, and EV‐related molecules such as CD63 offer new targets for early cancer diagnostics due to the EVs being aberrantly modified by cancer cells. EVs display and carry a multitude of different molecules to be analyzed for the development of new analytical methods sometimes referred to as liquid biopsies. The tetraspanin CD63 is linked to EVs and is overexpressed in BrCa in which its aberrant glycosylation has been reported to be mediated by ribophorin II.^15^ In this study, we found that CD63 and its possible complexes of EVs immobilized from BrCa cell line secretions were reactive with fucose‐binding lectins AAL and UEA. But, when these glycovariants were measured from serum, their levels did not differ between the healthy individuals and BrCa patients. Instead, the levels of total CD63, as measured with our experimental CD63 IA, were significantly different between the control and case groups and there was a clear indication that the CD63 IA could have some diagnostic ability for early stage BrCa according to ROC analysis (AUC = 0.835). Although the correlation between cell malignancy and the secretion rate of EVs is not clear, our results indicate that the overall quantity of CD63+ EVs is elevated in BrCa.^25^

The tumor marker CA125 measured from the mucin 16 glycoprotein is considered a valuable marker for ovarian cancer and its use is recommended for ovarian cancer screening in women with high risk.^26^ It has also been reported to be elevated in BrCa patients.^12^ The aberrant cancer‐related *O*‐glycosylation of mucins is generally considered to be based on core 1 glycosylation, meaning the T and Tn antigens^27^ and their sialylated derivatives.^28^ The antibodies against T and Tn antigens were tested for binding to CA125 derived from BrCa cell lines. The anti‐T antibody bound exceptionally well to CA125 from SKBR3 cell line. WGA was the other molecule that bound to the BrCa cell line materials, even more effectively than anti‐T antibody, and mostly to SKBR3. WGA is known to bind sialic acid and GlcNAc. GlcNAc is not present in the core 1 derived BrCa‐associated glycans but it is present in the core 2 derived glycans which have been associated with estrogen receptor negative (ER^−^) BrCas.^28^ The CA125^T^ and CA125^WGA^ assays were tested on serum samples and the CA125^T^ levels did not differ significantly between the controls and cases but the CA125^WGA^ levels did. The patient samples did not have the hormone receptor status available, so it is difficult to speculate whether the WGA bound to sialylated core 1 glycans or to the core 2 glycans that are commonly present in ER^−^ BrCas. The ROC analysis showed that the diagnostic ability of the CA125^WGA^ was good with an AUC of 0.814. The AUC of CA125^WGA^ was significantly better than the AUC of CA15‐3 IA which is currently the only FDA‐approved serological BrCa marker.

Multiple GBPs bound to BrCa‐associated CA15‐3 in the screening with cell line materials. The Concanavalin A (Con‐A) lectin reactive CA15‐3 levels have been found to distinguish between benign breast disease and BrCa in a different assay setup.^29^ Con‐A binds mannose structures that are generally present when *N*‐glycosylation is initiated so it was speculated that truncated *N*‐glycans of CA15‐3 might be the BrCa‐associated glycans that were detected in the assay.^29^ In our screening, Con‐A did not significantly bind to the CA15‐3 secreted by cultured BrCa cells. This might be due to the specific glycosylation machinery of the cells that were used in this study or even the culture conditions, which can affect the sensitive glycosylation process.^30^

Antibodies against the T and Tn antigens and the WGA bound most effectively, but MGL was also tested with clinical samples due to our previous promising results on metastatic BrCa samples.^18^ The GBPs bound CA15‐3 most likely due to the previously speculated reasons as why the GBPs bound to CA125, that is, the mucin‐related core 1 and/or core 2 *O*‐glycans. The GBPs that bound BrCa‐associated CA15‐3 in the screening were tested with the clinical samples. The CA15‐3^WGA^ levels were found to be statistically different between the controls and cases. The ROC analysis revealed that the AUC achieved by using the CA15‐3^WGA^ levels as a classifier was the highest of any of the tested assays with the value of 0.915. The AUC of CA15‐3^WGA^ was significantly higher than either of the conventional assays' AUC. The CA15‐3^WGA^ also distinguished metastatic BrCa patients from healthy in our previous study,^18^ so it is probably not limited to detecting localized BrCa.

Logistic regression models were also generated from the combination of the best‐performing glycovariant assays and the probabilities were plotted in Figure 5. The combination of CA15‐3^WGA^ and CD63 IA classified several BrCa samples correctly which were incorrectly classified as negatives (probability <.5) with individual assays. Compared to CA125^WGA^, CA15‐3^WGA^, and CD63 IA, four more, four more, and two more samples were correctly classified with the combination, respectively. The combination of all glycovariant assays classified only one more BrCa sample correctly, but the probabilities of disease were very close with the models (0.48 vs. 0.51). This implies that the CA15‐3^WGA^ assay and CD63 IA might complement each other.

The MCF7 cell line is an estrogen and progesterone positive (ER^+^, PR^+^) and rather well‐differentiated, poorly invasive adenocarcinoma cell line with low metastatic potential. The T47D cell line is derived from a metastatic ductal breast carcinoma. T47D is ER^+^ and PR^+^ and androgen receptor positive cell line T47D and is tumorigenic in mice. SKBR3 cell line is an HER2 overexpressing, ER and PR negative adenocarcinoma cell line‐, and it forms poorly differentiated tumors in mice.

The CD63^UEA^ was elevated only in the MCF7, which is the least aggressive of the used cell lines. It was derived from a postmenopausal patient, may explain the assay's poor performance in the differential diagnosis of the samples from premenopausal breast cancer patients, who often have aggressive tumors. The CA125^WGA^ was elevated in only SKBR3, which implies that this marker is elevated in poorly differentiated, hormone receptor negative adenocarcinomas. The assay that performed best for the differential diagnosis of the samples, CA15‐3^WGA^, was elevated most in MCF7 and T47D cell lines and not much in SKBR3 cell line. It seems that this glycovariant could be elevated with the hormone receptor positive, less aggressive adenocarcinoma. Even though two of the glycovariants found performed well in the differential diagnosis of the samples used, in the future a screening with an increased number of different breast cancer cell lines combined with a larger cohort of patients with more information on the breast cancer subtypes would best demonstrate the connections between the glycovariants and histopathological characteristics.

## 5 CONCLUSIONS

The major limitation of this study was the cohort size and the inability to correlate breast cancer subtypes with certain markers, and in the future these assays will be evaluated on larger cohorts and using relevant calibrators. Based on previous literature we hypothesized that glycosylation of proteins and EVs may provide targets for the early detection of BrCa, especially when coupled with a sensitive nanoparticle‐based approach and took it under examination. From the experimental data, we concluded that the levels of CD63, CA125^WGA^, and CA15‐3^WGA^ may be used to differentiate localized BrCa from healthy controls significantly better than the levels of conventional glycoprotein tumor markers CA125 and CA15‐3.

## CONFLICT OF INTEREST

The authors Kamlesh Gidwani and Kim Pettersson are inventors in a patent application “Lectin‐Based Diagnostics of Cancers” International Application No PCT/FI12017/050541.

## AUTHOR CONTRIBUTIONS

**Joonas Terävä:** Conceptualization (lead); data curation (lead); formal analysis (lead); funding acquisition (supporting); investigation (lead); methodology (lead); project administration (supporting); resources (lead); software (lead); supervision (lead); validation (lead); visualization (lead); writing & original draft (lead); writing & review and editing (lead). **Alejandra Verhassel:** Conceptualization (supporting); methodology (supporting); resources (equal); writing & review and editing (supporting). **Orsola Botti:** Conceptualization (supporting); formal analysis (supporting); investigation (supporting); writing & original draft (supporting); writing & review and editing (supporting). **Md. Khirul Islam:** Conceptualization (supporting); methodology (supporting); resources (equal); writing & review and editing (supporting). **Janne Leivo:** Conceptualization (lead); methodology (supporting); resources (supporting); writing & review and editing (supporting). **Pirkko Härkönen:** Conceptualization (supporting); resources (equal); writing & review and editing (supporting). **Kim Pettersson:** Conceptualization (lead); funding acquisition (lead); methodology (lead); project administration (lead); resources (lead); supervision (lead); writing & review and editing (supporting). **Kamlesh Gidwani:** Conceptualization (lead); methodology (lead); project administration (lead); resources (lead); supervision (lead); writing & review and editing (supporting).

## ETHICAL STATEMENT

The collection of the clinical samples was approved by Advarra IRB and another ethics committee. The other ethics committee's location information was redacted by Discovery Life Sciences.

## CONSENT TO PARTICIPATE

Written informed consent was obtained from all patients.

## CONSENT FOR PUBLICATION

Not applicable.

## Data Availability

The data that support the findings of this study are available from the corresponding author upon request.
